# *Vital Signs: *Drug Overdose Deaths, by Selected Sociodemographic and Social Determinants of Health Characteristics — 25 States and the District of Columbia, 2019–2020

**DOI:** 10.15585/mmwr.mm7129e2

**Published:** 2022-07-22

**Authors:** Mbabazi Kariisa, Nicole L. Davis, Sagar Kumar, Puja Seth, Christine L. Mattson, Farnaz Chowdhury, Christopher M. Jones

**Affiliations:** ^1^Division of Overdose Prevention, National Center for Injury Prevention and Control, CDC; ^2^Peers and Partners, Inc., Atlanta, Georgia; ^3^National Center for Injury Prevention and Control, CDC.

## Abstract

**Introduction:** Drug overdose deaths increased approximately 30% from 2019 to 2020 in the United States. Examining rates by demographic and social determinants of health characteristics can identify disproportionately affected populations and inform strategies to reduce drug overdose deaths.

**Methods:** Data from the State Unintentional Drug Overdose Reporting System (SUDORS) were used to analyze overdose death rates from 2019 to 2020 in 25 states and the District of Columbia. Rates were examined by race and ethnicity and county-level social determinants of health (e.g., income inequality and treatment provider availability).

**Results:** From 2019 to 2020, drug overdose death rates increased by 44% and 39% among non-Hispanic Black (Black) and non-Hispanic American Indian or Alaska Native (AI/AN) persons, respectively. Significant disparities were found across sex, age, and racial and ethnic subgroups. In particular, the rate in 2020 among Black males aged ≥65 years (52.6 per 100,000) was nearly seven times that of non-Hispanic White males aged ≥65 years (7.7). A history of substance use was frequently reported. Evidence of previous substance use treatment was lowest for Black persons (8.3%). Disparities in overdose deaths, particularly among Black persons, were larger in counties with greater income inequality. Opioid overdose rates in 2020 were higher in areas with more opioid treatment program availability compared with areas with lower opioid treatment availability, particularly among Black (34.3 versus 16.6) and AI/AN (33.4 versus 16.2) persons.

**Conclusions and Implications for Public Health Practice:** Health disparities in overdose rates continue to worsen, particularly among Black and AI/AN persons; social determinants of health, such as income inequality, exacerbate these inequities. Implementation of available, evidence-based, culturally responsive overdose prevention and response efforts that address health disparities impacting disproportionately affected populations are urgently needed.

## Introduction

The 91,799 drug overdose deaths that occurred in the United States in 2020 represent an approximately 30% increase from 2019 (*1*). The COVID-19 pandemic and disruption in access to prevention, treatment, and harm reduction services have likely contributed to this increase (*2*). Recent increases in drug overdose deaths were largely driven by illicitly manufactured fentanyl and fentanyl analogs (collectively referred to as IMFs) (*1*,*3*,*4*). Deaths involving stimulants, such as cocaine and psychostimulants with abuse potential, (e.g., methamphetamine) also increased in recent years and often co-occurred with opioids (*1*,*3*,*5*,*6*); some racial and ethnic minority groups were disproportionately affected (*6*).

Disparities in overdose mortality rates are not fully explained by substance use patterns (*7*,*8*) and might result from unequal access to substance use treatment services (*9*), socioeconomic inequities, and social determinants of health (*10*). Non-Hispanic Black (Black) and non-Hispanic American Indian or Alaska Native (AI/AN) persons report barriers to accessing mental health services and substance use treatment (*9*). However, the impact of treatment access and income inequality on drug overdose mortality has not been fully explored, particularly during the COVID-19 pandemic, which exacerbated disparities (*11*).

This report describes changes in drug overdose death rates from 2019 to 2020, stratified by sex, age group, and race and ethnicity. In addition, it examines differences in circumstances surrounding drug overdose, and assesses differences in overdose death rates by county-level income inequality and availability of mental health treatment providers and providers of medications for opioid use disorder.

## Methods

Data on drug overdose deaths of unintentional and undetermined intent during 2019–2020 were obtained from the State Unintentional Drug Overdose Reporting System (SUDORS). This system includes information collected from death certificates and medical examiner or coroner reports (e.g., full postmortem toxicology results and death scene investigation findings).* Analyses were limited to 26 jurisdictions (25 states and the District of Columbia [DC]) that submitted complete 2019–2020 data.^†^ Death rates (overdose deaths per 100,000 population) were age-adjusted to the 2000 U.S. standard population, and rate ratios were calculated.^§^ U.S. Census Bureau bridged-race population estimates were assessed for the following racial and ethnic groups: non-Hispanic Asian or Pacific Islander (A/PI), AI/AN, Black, non-Hispanic White (White), and Hispanic persons. Rates based on <20 deaths and counts <10 were suppressed.

Information on income inequality and mental health provider availability (number of mental health providers per 100,000 population) was obtained from the 2021 County Health Rankings and analyzed by tertile.^¶^ The Drug Enforcement Administration’s controlled substance registration database was used to ascertain whether a county had at least one opioid treatment program and to estimate Drug Addiction Treatment Act of 2000 (DATA)-waived provider capacity (qualified clinicians who can prescribe buprenorphine in office-based settings for opioid use disorder treatment) by county.**

Differences in age-adjusted death rates from 2019 to 2020 were considered statistically significant if CIs did not overlap; a gamma distribution was used if <100 deaths occurred in either year.^††^ Analyses were conducted using SAS (version 9.4; SAS Institute). This activity was reviewed by CDC and was conducted consistent with applicable federal law and CDC policy.^§§^

## Results

From 2019 to 2020, overall drug overdose death rates increased in 25 states and DC; the largest increases occurred among certain racial/ethnic minority populations. Relative rate increases were highest among Black (44%) and AI/AN persons (39%) (Table 1). Among White persons, the rate increased by 22%. Within racial/ethnic groups, overdose death rates also varied by age. Black persons aged 15–24 years experienced the largest relative rate increase from 2019 to 2020 (86%). Among AI/AN persons, the highest relative rate increase occurred among those aged 25–44 years (49%). Among White persons, those aged 15–24 years experienced the largest relative rate increase (34%).

**TABLE 1:** Annual number and age-adjusted rate of drug overdose deaths,* by age and race and Hispanic origin^†^ — 25 states and the District of Columbia,^§^ 2019–2020

Race and ethnicity/Age group, yrs	No. (rate)		Absolute change^¶^	Relative change (%)^¶^	Rate ratio**	
	2019	2020			2019	2020
**White**						
**All ages**	**21,921 (25.2)**	**26,625 (30.7)**	**5.5^††^**	**22^††^**	**Ref**	**Ref**
15–24	1,315 (12.3)	1,749 (16.5)	4.2^††^	34^††^	Ref	Ref
25–44	11,641 (52.3)	14,016 (62.7)	10.4^††^	20^††^	Ref	Ref
45–64	8,187 (32.9)	9,901 (40.5)	7.6^††^	23^††^	Ref	Ref
≥65	761 (4.3)	932 (5.1)	0.8^††^	19^††^	Ref	Ref
**Black**						
**All ages**	**5,146 (27.0)**	**7,467 (38.9)**	**11.9^††^**	**44^††^**	**1.07**	**1.27**
15–24	221 (7.8)	411 (14.5)	6.7^††^	86^††^	0.63	0.88
25–44	1,891 (35.4)	2,972 (54.7)	19.3^††^	55^††^	0.68	0.87
45–64	2,626 (58.5)	3,477 (77.6)	19.1^††^	33^††^	1.78	1.92
≥65	390 (17.8)	587 (25.7)	7.9^††^	44^††^	4.14	5.04
**AI/AN**						
**All ages**	**327 (26.2)**	**456 (36.4)**	**10.2^††^**	**39^††^**	**1.04**	**1.19**
15–24	28 (14.4)	31 (16.0)	1.6	11	1.17	0.97
25–44	179 (50.5)	270 (75.1)	24.6^††^	49^††^	0.97	1.20
45–64	107 (36.1)	145 (49.3)	13.2	37	1.10	1.22
≥65	13^§§^	—^§§^	—^§§^	—^§§^	—^§§^	—^§§^
**A/PI**						
**All ages**	**203 (2.7)**	**252 (3.3)**	**0.6**	**22**	**0.11**	**0.11**
15–24	27 (2.9)	31 (3.3)	0.4	14	0.24	0.20
25–44	136 (5.7)	160 (6.6)	0.9	16	0.11	0.11
45–64	37 (2.3)	55 (3.3)	1.0	43	0.07	0.08
≥65	—^§§^	—^§§^	—^§§^	—^§§^	—^§§^	—^§§^
**Hispanic**						
**All ages**	**2,473 (17.3)**	**3,081 (21.0)**	**3.7^††^**	**21^††^**	**0.69**	**0.68**
15–24	209 (8.3)	323 (12.5)	4.2^††^	51^††^	0.67	0.76
25–44	1,399 (30.7)	1,716 (37.1)	6.4^††^	21^††^	0.59	0.59
45–64	812 (28.5)	965 (32.7)	4.2	15	0.87	0.81
≥65	49 (5.2)	76 (7.6)	2.4	46	1.21	1.49

**Source:** State Unintentional Drug Overdose Reporting System.

**Abbreviations:** A/PI = Asian or Pacific Islander; AI/AN = American Indian or Alaska Native; Ref = referent group.

* Rates are age-adjusted using the direct method and the 2000 U.S. standard population, except for age-specific crude rates. All rates are deaths per 100,000 population.

^†^ A/PI, AI/AN, Black, and White persons are non-Hispanic; Hispanic persons could be of any race. Data for Hispanic origin should be interpreted with caution; studies comparing Hispanic origin on death certificates and on U.S. Census Bureau surveys have shown inconsistent reporting on Hispanic ethnicity. Potential race misclassification might lead to underestimates for certain categories, primarily non-Hispanic A/PI and non-Hispanic AI/AN decedents. https://www.cdc.gov/nchs/data/series/sr_02/sr02_172.pdf

^§^ Includes 26 jurisdictions with complete data in 2019 and 2020: Alaska, Connecticut, Delaware, District of Columbia, Georgia, Illinois, Kentucky, Maine, Massachusetts, Minnesota, Missouri, Nevada, New Hampshire, New Jersey, New Mexico, North Carolina, Ohio, Oklahoma, Pennsylvania, Rhode Island, Tennessee, Utah, Vermont, Virginia, Washington, and West Virginia.

^¶^ Absolute rate change is the difference between 2019 and 2020 rates. Relative change is the absolute rate change divided by the 2019 rate, multiplied by 100.

** Rate ratio is calculated by dividing the rate for persons of race/ethnicities other than White by the rate for White persons. For example, the 2019 rate ratio for Black persons is determined by dividing their rate in 2019 by the rate of White persons in that same year.

^††^ Statistically significant (p value <0.05). Nonoverlapping CIs were used to assess statistical significance between 2019 and 2020. The method of comparing CIs is a conservative method for statistical significance; caution should be observed when interpreting a nonsignificant difference when the lower and upper limits being compared overlap only slightly.

^§§^ Cells with nine or fewer deaths are not reported. Rates based on <20 deaths are not considered reliable and are not reported.

When stratified by sex and age group, higher overdose death rates occurred among older Black males, with the highest rate in 2020 among those aged 45–64 years (124.9) (Supplementary Table, https://stacks.cdc.gov/view/cdc/118656). In addition, rates among Black males aged ≥65 years were nearly six times as high as those among White males of the same age in 2019 (35.7 versus 6.2), increasing to nearly seven times as high in 2020 (52.6 versus 7.7). Among AI/AN males, those aged 25–44 years experienced the highest rates in 2019 (67.5) and 2020 (87.2), similar to rates among White males in this age group (2019 = 72.7; 2020 = 87.0). Among Hispanic males, those aged 25–44 years had the highest rates in 2019 (47.6) and 2020 (57.3). The rate for Hispanic males aged 15–24 years increased 47% from 12.9 in 2019 to 18.9 in 2020.

Among females, the largest rate disparities between AI/AN and White decedents were observed among those aged 25–44 years, with the disparity increasing nearly 57% from 2019 to 2020 (2019 rate ratio = 1.06; 2020 rate ratio = 1.66). AI/AN females aged 25–44 years also had the largest relative increase in overdose death rate from 2019 to 2020 (88%).

A documented history of substance use was commonly reported for most decedents, with the highest proportion among White (78.3%), AI/AN (77.4%), and Hispanic (74.8%) decedents (Table 2). However, the proportion of decedents with documented evidence of previous substance use treatment was low overall, with the lowest proportions among Black (8.3%), Hispanic (10.2%), and AI/AN (10.7%) decedents. Evidence of injection drug use was most prevalent among White (28.0%) and AI/AN (22.9%) decedents. Evidence of naloxone administration was highest among AI/AN (21.5%) decedents and lowest among A/PI (16.4%) decedents but was low in all groups.

**TABLE 2:** Characteristics of drug overdose deaths, overall and by race and Hispanic origin*^,^^†^ — 25 states and the District of Columbia,^§^ 2019–2020

Characteristic^¶^	No. (%)					
	White	Black	AI/AN	A/PI	Hispanic	Total
Other substance use problem**	37,128 (78.3)	9,127 (74.0)	603 (77.4)	320 (71.0)	4,119 (74.8)	**52,052 (77.2)**
**Treatment for substance use/misuse** ^††^	7,780 (16.4)	1,024 (8.3)	82 (10.7)	58 (12.9)	560 (10.2)	**9,621 (14.3)**
**Bystander present**	19,460 (41.0)	5,259 (42.7)	413 (53.0)	186 (41.2)	2,475 (44.9)	**28,246 (41.9)**
**Naloxone administered**	9,353 (19.7)	2,501 (20.3)	166 (21.5)	74 (16.4)	1,025 (18.6)	**13,311 (19.8)**
**Recent relapse**	3,895 (8.2)	424 (3.4)	48 (6.2)	18 (4.0)	350 (6.4)	**4,793 (7.1)**
**Previous overdose**	5,489 (11.6)	1,094 (8.9)	80 (10.3)	26 (5.8)	476 (8.6)	**7,256 (10.8)**
**Recent release from jail**	1,497 (3.2)	400 (3.2)	30 (3.9)	—^§§^	215 (3.9)	**2,173 (3.2)**
**Current treatment for pain**	4,453 (9.4)	810 (6.6)	65 (8.4)	20 (4.4)	293 (5.3)	**5,709 (8.5)**
**Evidence of injection**	13,255 (28.0)	1,366 (11.1)	177 (22.9)	77 (17.1)	1,075 (19.5)	**16,188 (24.0)**

**Source:** SUDORS.

**Abbreviations:** A/PI = Asian or Pacific Islander; AI/AN = American Indian or Alaska Native; SUDORS = State Unintentional Drug Overdose Reporting System.

* Totals include persons who are multiracial or have an unknown race and ethnicity.

^†^ A/PI, AI/AN, Black, and White persons were non-Hispanic. Hispanic persons could be of any race. Data for Hispanic origin should be interpreted with caution; studies comparing Hispanic origin on death certificates and on U.S. Census Bureau census surveys have shown inconsistent reporting on Hispanic ethnicity. Potential race misclassification might lead to underestimates for certain categories, primarily non-Hispanic A/PI and non-Hispanic AI/AN decedents. https://www.cdc.gov/nchs/data/series/sr_02/sr02_172.pdf

^§^ Includes 26 jurisdictions with complete data in 2019 and 2020: Alaska, Connecticut, Delaware, District of Columbia, Georgia, Illinois, Kentucky, Maine, Massachusetts, Minnesota, Missouri, Nevada, New Hampshire, New Jersey, New Mexico, North Carolina, Ohio, Oklahoma, Pennsylvania, Rhode Island, Tennessee, Utah, Vermont, Virginia, Washington, and West Virginia. Analysis of circumstance data was limited to cases with a medical examiner/coroner report and focused primarily on the most common characteristics of drug overdose deaths. Data for July–December 2020 for Tennessee were not included because the overall percentage of decedents with a medical examiner or coroner report was <75%, which is the cutoff used in SUDORS for inclusion in analyses of overdose circumstances.

^¶^ Missing values were excluded from calculations of percentages. Percentages might not sum to 100% because of rounding. A total of 445 decedents were of an unknown race/ethnicity.

** Includes documented evidence of a substance use disorder for substances other than alcohol.

^††^ Includes documented evidence of past or current substance use disorder treatment.

^§§^ Cells with nine or fewer deaths are not reported.

**FIGURE 1:**
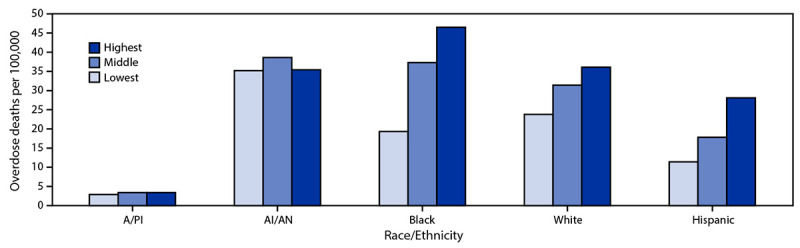
Age-adjusted rates* of drug overdose deaths, by race/ethnicity^†^ and income inequality ratio^§^ — 25 states and the District of Columbia,^¶^ 2020 **Abbreviations:** A/PI = Asian or Pacific Islander; AI/AN = American Indian or Alaska Native. * Rates (overdose deaths per 100,000 population) age-adjusted to the 2000 U.S. standard population using the vintage year population of the data year. ^†^ A/PI, AI/AN, Black, and White persons are non-Hispanic; Hispanic persons could be of any race. Data for Hispanic origin should be interpreted with caution; studies comparing Hispanic origin on death certificates and on U.S. Census Bureau surveys have shown inconsistent reporting on Hispanic ethnicity. Potential race misclassification might lead to underestimates for certain categories, primarily non-Hispanic A/PI and non-Hispanic AI/AN decedents. https://www.cdc.gov/nchs/data/series/sr_02/sr02_172.pdf ^§^ The 2021 County Health Rankings used data from the 2015–2019 American Community Survey for the income inequality ratio. Income inequality is defined as the ratio of household income at the 80th percentile to income at the 20th percentile (i.e., when the incomes of all households in a county are listed from highest to lowest, the 80th percentile is the level of income at which only 20% of households have higher incomes, and the 20th percentile is the level of income at which only 20% of households have lower incomes). A higher inequality ratio indicates greater division between the top and bottom ends of the income spectrum. The specific ranges for income inequality groups are defined as lowest (2.7–4.1), middle (4.2–4.7), and highest (4.8–10.5). ^¶^ Alaska, Connecticut, Delaware, District of Columbia, Georgia, Kentucky, Maine, Massachusetts, Minnesota, Nevada, New Hampshire, New Jersey, New Mexico, North Carolina, Ohio, Oklahoma, Rhode Island, Tennessee, Utah, Vermont, Virginia, and West Virginia were funded to report cause of death data on all overdose deaths within the jurisdiction in 2019 and 2020. Illinois, Missouri, Pennsylvania, and Washington were funded to report cause of death data on ≥75% of all overdose deaths within a jurisdiction in 2019 and 2020. Jurisdictions were included in rate calculations if they met data submission deadlines and addressed data entry errors in 2019 and 2020.

In 2020, overdose death rates increased with increasing county-level income inequality ratios (the ratio of household income at the 80th percentile to income at the 20th percentile) across most racial/ethnic groups, but Black and Hispanic persons were disproportionally affected (Figure 1). Among Black persons, the overdose rate for counties with the highest income inequality (46.5) was more than twice that of counties with the lowest income inequality (19.3). In counties with the lowest income inequality, the rate was highest among AI/AN persons (35.2); in counties with the highest income inequality, the rate was highest among Black persons (46.5). Among Hispanic persons, the overdose rate in counties with the highest income inequality (28.1) was more than twice that of counties with the lowest income inequality (11.4).

Drug overdose death rates were higher in counties with a higher potential capacity for treatment of mental health conditions (based on mental health provider availability), and this varied by race and ethnicity. Among Black persons, the drug overdose rate during 2020 in areas with the highest mental health provider availability (46.7) was more than 2.5 times as high as the rate in areas with the lowest rate of providers (17.2) (Supplementary Figure 1, https://stacks.cdc.gov/view/cdc/118654).

**FIGURE 2:**
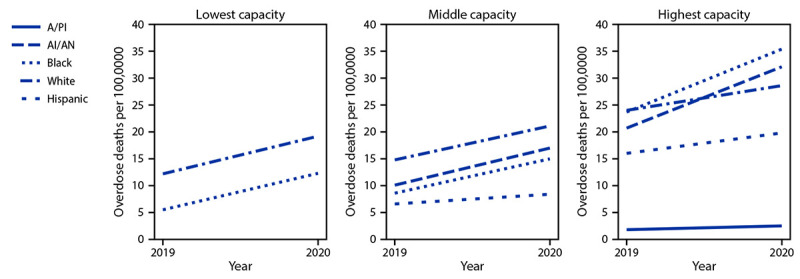
Changes in age-adjusted* rates^†^ of opioid overdose deaths, by race/ethnicity^§^ and Drug Addiction Treatment Act–waived provider capacity^¶^ tertile — 25 states and the District of Columbia,** 2019–2020 **Abbreviations:** A/PI = Asian or Pacific Islander; AI/AN = American Indian or Alaska Native; DATA = Drug Addiction Treatment Act. * Rates (overdose deaths per 100,000 population) age-adjusted to the 2000 U.S. standard population using the vintage year population of the data year. ^†^ Rates based on <20 deaths are not considered reliable and not reported. This suppression rule applied to A/PI and AI/AN persons in the lowest-capacity tertile as well as A/PI persons in the medium-capacity tertile for 2019 and 2020. The suppression rule also applied to Hispanic persons in the lowest-capacity tertile in 2019; however, the age-adjusted rate for Hispanic persons in 2020 (8.9 per 100,000) was not presented because it could not be compared with a 2019 rate. ^§^ A/PI, AI/AN, Black, and White persons are non-Hispanic; Hispanic persons could be of any race. Data for Hispanic origin should be interpreted with caution; studies comparing Hispanic origin on death certificates and on U.S. Census Bureau surveys have shown inconsistent reporting of Hispanic ethnicity. Potential race misclassification might lead to underestimates for certain categories, primarily non-Hispanic A/PI and non-Hispanic AI/AN decedents. https://www.cdc.gov/nchs/data/series/sr_02/sr02_172.pdf ^¶^ In 2000, DATA granted waivers to qualified physicians to prescribe buprenorphine in in-office settings for opioid use disorder treatment. In 2016, the Comprehensive Addiction and Recovery Act permitted nurse practitioners and physician assistants to obtain DATA waivers to prescribe buprenorphine. DATA-waived clinicians can provide office-based opioid treatment to 30, 100, or 275 patients at a given time. Potential treatment capacity was calculated by multiplying the number of DATA-waived providers by their maximum patient limit (30, 100, or 275 patients) and presented by tertile. The specific ranges for DATA-waived provider capacity are lowest capacity (0–119), middle capacity (120–769), and highest capacity (770–64,105). ** Alaska, Connecticut, Delaware, District of Columbia, Georgia, Kentucky, Maine, Massachusetts, Minnesota, Nevada, New Hampshire, New Jersey, New Mexico, North Carolina, Ohio, Oklahoma, Rhode Island, Tennessee, Utah, Vermont, Virginia, and West Virginia were funded to report cause of death data on all overdose deaths within the jurisdiction in 2019 and 2020. Illinois, Missouri, Pennsylvania, and Washington were funded to report cause of death data on ≥75% of all overdose deaths within a jurisdiction in 2019 and 2020. Jurisdictions were included in rate calculations if they met data submission deadlines and addressed data entry errors in 2019 and 2020.

In 2020, the rates of opioid-involved deaths among Black and AI/AN persons in counties with at least one opioid treatment program were more than twice those in counties without opioid treatment programs (Black = 34.3 versus 16.6; AI/AN = 33.4 versus 16.2) (Supplementary Figure 2, https://stacks.cdc.gov/view/cdc/118655). In addition, the opioid-involved death rate among Black persons in counties with higher potential buprenorphine capacity from DATA-waived providers (35.4) was nearly triple that in counties with low potential capacity (12.3). Among counties with higher potential treatment capacity, overdose death rates increased 49% among Black persons from 2019 (23.7) to 2020 (35.4) and 55% among AI/AN persons (from 20.7 to 32.1) compared with 19% among White persons (from 24.0 to 28.6) (Figure 2).

## Discussion

This study highlights five critical findings on health disparities and inequities related to drug overdose deaths in the United States. First, from 2019 to 2020, disproportionate increases occurred among Black (44%) and AI/AN (39%) persons compared with those among White persons (22%). Among demographic subgroups, the rate among Black males aged ≥65 years increased to nearly seven times that of White males of the same age, and the rate among AI/AN females aged 25–44 years increased to nearly twice that of White females of the same age in 2020. Second, drug overdose death rates increased with increasing county-level income inequality, particularly among Black persons, among whom the overdose death rate was more than twice as high in areas with the highest income inequality as in areas with the lowest income inequality. Third, evidence of previous substance use treatment was lowest among Black decedents and approximately one half that of White decedents. Fourth, overdose death rates were highest in counties with higher potential substance use treatment capacity and mental health providers, and rates were more pronounced among Black and AI/AN persons than among White persons, likely associated with long-standing inequities in access to mental health and substance use care, including medications for opioid use disorder. Finally, evidence of naloxone administration was highest among AI/AN (21.5%) decedents and lowest among A/PI (16.4%) decedents but was low in all groups. These findings can help guide the implementation of equitable overdose prevention and response efforts.

Prioritizing prevention and substance use disorder treatment for persons in areas with higher economic inequities is particularly important for certain groups. Higher drug use has been reported in areas with more economic distress, which increases the risk for fatal overdose (*12*). Further, impacts of income inequality (e.g., housing instability, transportation access, and insurance status), long-standing mistrust in the health care system, stigma, and bias contribute to treatment access barriers (*12*–*14*). In this analysis, Black decedents were the least likely racial or ethnic group to have evidence of substance use treatment, and 2020 overdose rates were highest among Black and AI/AN persons in areas with high treatment provider availability. Although high-prevalence areas might have a greater proportion of treatment services, this higher potential treatment capacity might not reflect treatment services that are accessible to community members, especially in counties that cover large geographic areas. The clustering of providers in denser population centers could result in transportation barriers for persons residing in less populated areas of the county.^¶¶^ Structural and policy-level interventions are essential to address these access barriers. These include expanding linkage to and retention in care, equitable access to treatment (e.g., medication for opioid use disorder) and behavioral health interventions, and harm reduction services (e.g., naloxone, comprehensive syringe services programs, and fentanyl test strips).

The COVID-19 pandemic has highlighted long-neglected disparities in access to and provision of health care among AI/AN, Black, and Hispanic persons (*11*). The findings in this report underscore the increasing impact of the escalating overdose crisis on these populations. More stigmatization, criminalization, and lack of access to evidence-based treatments among racial/ethnic minority groups with substance use disorders have been well-documented (*15*). These barriers might further elucidate the disparities observed in reported history of substance use treatment and overdose death rates by income inequality and mental health provider availability among Black and AI/AN persons. For example, Black persons have more limited access to buprenorphine treatment than do White persons, and in AI/AN communities underfunding of tribal clinics has affected the availability of mental health treatment (*9*,*14*). In addition, polysubstance use and the increasing proliferation of IMFs in the drug supply have exacerbated the surge in overdose deaths (*5*). 

Prevention efforts must rapidly incorporate existing, evidence-based, culturally responsive interventions that address polysubstance use and social determinants of health to reduce inequities around prevention, treatment, and harm reduction. Integration of evidence-based substance use disorder treatment with culturally tailored traditional practices, spirituality, and religion might improve treatment acceptance among Black and AI/AN populations (*16**,**17*,*18*). Culturally specific awareness campaigns, employment in nontraditional and community settings, and trusted community prevention messengers to assist with linkages to treatment and harm reduction services could reduce stigma and mistrust as well as improve access and provision of care (*18*,*19*). In addition, expanding the current evidence base to address upstream drivers of inequity and implementing primary prevention efforts that focus on adverse childhood experiences that predispose persons to risk for substance use and substance use disorder as well as implementing trauma-informed care and services are critical.***

The findings in this report are subject to at least four limitations. First, analyses were limited to 26 jurisdictions reporting data to SUDORS, do not include all overdose deaths in the United States, and might not be generalizable. Second, overdose circumstance data are limited to information provided in investigative reports; therefore, overdose risk factors might be underestimated. Third, potential race and ethnicity misclassification might underestimate rates for certain populations, primarily Hispanic, AI/AN, and A/PI persons.^†††^ Finally, because of low counts, rates for multiracial groups were not included in analyses.

Provisional estimates indicate continued increases in drug overdose deaths in 2021 (*20*). Health disparities and inequities are likely exacerbating these increases, particularly among racial/ethnic minority groups. Drug overdoses are preventable, and rapidly scaling up multisectoral, culturally responsive prevention efforts across federal, state, local, and tribal entities that place equity as a central tenet to address the escalating overdose crisis is urgently needed.

**Summary**

**What is already known about this topic?**

Drug overdose deaths increased 30% in the United States from 2019 to 2020. Known health disparities exist in overdose mortality rates, particularly among certain racial/ethnic minority populations.

**What is added by this report?**

From 2019 to 2020, overdose death rates increased by 44% and 39% among non-Hispanic Black (Black) and non-Hispanic American Indian or Alaska Native persons, respectively. As county-level income inequality increased, overdose rates increased, particularly among Black persons. Evidence of previous substance use treatment was lowest for Black decedents (8.3%).

**What are the implications for public health practice?**

Implementation of an evidence-based, culturally responsive, multisectoral approach is critical to reducing disparities in overdose rates. This includes addressing structural barriers and enhancing efforts such as linkage to care and harm reduction services.

## Footnotes

* SUDORS began in 2016 as part of CDC’s Enhanced State Opioid Overdose Surveillance (ESOOS) program, which funded 12 states, with an additional 20 states and DC funded in 2017 to abstract data on opioid overdose deaths. In 2019, SUDORS expanded to collect data on all drug overdose deaths from 47 states and DC (collectively referred to as jurisdictions) as part of CDC’s Overdose Data to Action (OD2A) program. https://www.cdc.gov/drugoverdose/od2a/index.html

† Alaska, Connecticut, DC, Delaware, Georgia, Kentucky, Maine, Massachusetts, Minnesota, Nevada, New Hampshire, New Jersey, New Mexico, North Carolina, Ohio, Oklahoma, Rhode Island, Tennessee, Utah, Vermont, Virginia, and West Virginia were funded to report cause of death data on all overdose deaths within the jurisdiction in 2019 and 2020. Illinois, Missouri, Pennsylvania, and Washington were funded to report cause of death data on ≥75% of all overdose deaths within a jurisdiction in 2019 and 2020. Jurisdictions were included in rate calculations if they met data submission deadlines and addressed data entry errors in 2019 and 2020. The analysis of circumstance data was limited to jurisdictions with medical examiner/coroner information and focused primarily on the most common characteristics of drug overdose deaths. Data for July–December 2020 for Tennessee were not included because the overall percentage of decedents with a medical examiner or coroner report was <75%, which is the cutoff used in SUDORS for inclusion in analyses of overdose circumstances. There were <1% of decedents with an unknown race/ethnicity.

§ Rates (deaths per 100,000 population) were age-adjusted to the 2000 U.S. standard population using the vintage year population of the data year. Rate ratios were calculated by dividing the rate for persons who were not White by the rate for White persons. For example, the 2019 rate ratio for Black persons was determined by dividing their rate in 2019 by the rate of White persons in that same year. Rates were based on occurrent deaths and resident population. Persons might possibly not have resided in the states where they died; however, a sensitivity analysis showed that in 2020, >95% of overdose deaths occurred in the state where the decedent resided.

¶ The 2021 County Health Rankings used data from the 2015–2019 American Community Survey for the income inequality ratio. Income inequality is defined as the ratio of household income at the 80th percentile to income at the 20th percentile (i.e., when the incomes of all households in a county are listed from highest to lowest, the 80th percentile is the level of income at which only 20% of households have higher incomes, and the 20th percentile is the level of income at which only 20% of households have lower incomes). A higher inequality ratio indicates greater division between the top and bottom ends of the income spectrum. The specific ranges for income inequality groups are defined as low-income inequality (2.7–4.1), middle-income inequality (4.2–4.7), and high-income inequality (4.8–10.5). The 2021 County Health Rankings used 2020 data from the Centers for Medicare & Medicaid Services National Provider Identification registry for the number of mental health providers.

** In 2000, DATA granted waivers to qualified physicians to prescribe buprenorphine in in-office settings for opioid use disorder treatment. In 2016, the Comprehensive Addiction and Recovery Act permitted nurse practitioners and physician assistants to obtain DATA waivers to prescribe buprenorphine. DATA-waived clinicians can provide office-based opioid treatment to 30, 100, or 275 patients at a given time. Potential treatment capacity was calculated by multiplying the number of DATA-waived providers by their maximum patient limit (30, 100, or 275 patients) and presented by tertile. The ranges for DATA-waived provider capacity are lowest capacity (0–119), middle capacity (120–769), and highest capacity (770–64,105).

†† Absolute rate change is the difference between 2019 and 2020 rates. Relative rate change is the absolute rate change divided by the 2019 rate, multiplied by 100.

§§ 45 C.F.R. part 46.102(l)(2), 21 C.F.R. part 56; 42 U.S.C. Sect. 241(d); 5 U.S.C. Sect. 552a; 44 U.S.C. Sect. 3501 et seq.

¶¶
https://oig.hhs.gov/oei/reports/oei-12-17-00240.pdf

***
https://www.cdc.gov/violenceprevention/pdf/preventingACES.pdf

††† The categorization of race and ethnicity in SUDORS data was limited to the following classifications: American Indian or Alaska Native, Asian or Pacific Islander, Black, White, Hispanic, Multiracial, and Other. Separating the Hispanic category into different racial groups was not performed because it would yield small numbers and the inability to calculate stable rates.
